# Proteasome inhibitors: Their effects on arachidonic acid release from cells in culture and arachidonic acid metabolism in rat liver cells

**DOI:** 10.1186/1471-2210-4-15

**Published:** 2004-08-05

**Authors:** Lawrence Levine

**Affiliations:** 1 Department of Biochemistry, Brandeis University Waltham, MA 02454, USA

## Abstract

**Background:**

I have postulated that arachidonic acid release from rat liver cells is associated with cancer chemoprevention. Since it has been reported that inhibition of proteasome activities may prevent cancer, the effects of proteasome inhibitors on arachidonic acid release from cells and on prostaglandin I_2 _production in rat liver cells were studied.

**Results:**

The proteasome inhibitors, epoxomicin, lactacystin and carbobenzoxy-leucyl-leucyl-leucinal, stimulate the release of arachidonic acid from rat glial, human colon carcinoma, human breast carcinoma and the rat liver cells. They also stimulate basal and induced prostacycin production in the rat liver cells. The stimulated arachidonic acid release and basal prostaglandin I_2 _production in rat liver cells is inhibited by actinomycin D.

**Conclusions:**

Stimulation of arachidonic acid release and arachidonic acid metabolism may be associated with some of the biologic effects observed after proteasome inhibition, e.g. prevention of tumor growth, induction of apoptosis, stimulation of bone formation.

## Background

The proteasome degrades many cellular proteins, several with regulatory functions. It is not surprising that proteasome inhibitors affect many biologic processes [1] including prevention of cancer [2]. The effect of proteasome inhibition on cell growth and possible cancer chemoprevention has been reviewed by Adams [3].

Epoxomicin, an α'-β'-epoxyketone, appears to be the most selective proteasome inhibitor. Based on its anti-tumor activity, this product was originally isolated from an actinomycetes strain C-996-17 [4]. It inhibits the chymotrypsin-like activity (cleavage after large hydrophobic residues), trypsin-like activity (cleavage after basic residues) and peptidyl-glutamyl peptide hydrolyzing (PGPH) activity (cleavage after acidic residues) of proteasomes. Activities of the Ca^++^-dependent proteases, calpain, papain, chymotrypsin, trypsin and cathepsin are not affected by epoxomicin even at a 50 μM concentration [5].

The β-lactone, lactacystin, is relatively selective but can inhibit cathepsin A [6]. Peptide aldehydes, in addition to inhibiting proteasome activity, can also inhibit lysosomal and Ca^++^-activated proteases [7]. The peptides containing the carboxyvinylsulfone moiety inhibit cysteine proteases [8,9].

I have shown that inhibition of proteolysis by phenylmethylsulphonyl fluoride, the peptide aldehydes carbobenzoxy-leucyl-leucyl-norvalinal and carbobenzoxy-leucyl-leucyl-leucinal (ZLLL) and lactacystin stimulate induced prostaglandin (PGI_2_) production in rat liver cells [10,11]. Lactacystin stimulates arachidonic acid (AA) release from these cells [11]. Others have reported that proteasome inhibition up-regulates cyclooxygenase-2 (COX-2) and stimulates PGE_2 _production in neuronal cells [12].

In this report, evidence is presented that proteasome inhibitors stimulate PGI_2 _production by rat liver cells as well as the release of AA from rat liver, rat glial, human colon carcinoma and human breast carcinoma cells in culture. The stimulation of AA release from rat liver cells is partially inhibited by preincubation of the cells with actinomycin D.

## Results and Discussion

**Figure 1:**
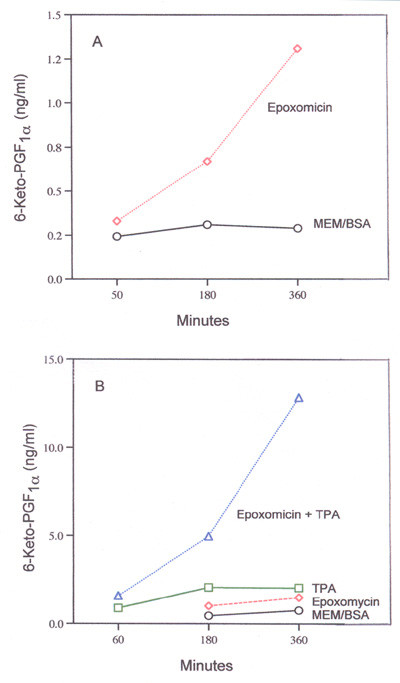
Time-course of (A) basal and (B) TPA-induced 6-keto-PGF_1α _production during incubation with 1.2 μM epoxomicin. In (B) 16.7 nM TPA was used. Analyses were performed with duplicate and triplicate dishes. Mean values are shown.

Of the cells examined (C-9 rat liver, C-6 rat glial, HT-29 human colon carcinoma and BT-20 human breast carcinoma) the prostanoid metabolic profile has been described only for C-9 rat liver cells (95% is PGI_2 _and less than 5% is PGE_2 _and PGF_2α_) [13]. At the low cell densities used in this study, only PGI_2_, the main product of COX-mediated synthesis, can be quantitatively estimated. The rat liver cells express COX-2 both constitutively and after induction [14]. The effect of time on basal and 12-0-tetradecanoylphorbol-13-acetate (TPA) induced PGI_2 _synthesis during incubation of cells with epoxomicin is shown in Fig. 1.

**Figure 2:**
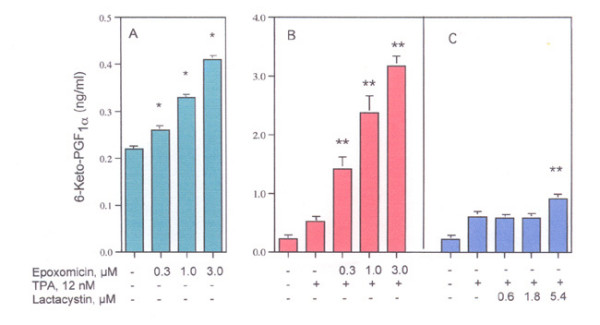
Effect of epoxomicin on (A) basal and (B) TPA-induced 6-ketoPGF_1α _production and (C) effect of lactacystin on TPA-induced 6-ketoPGF_1α _production. Cells were incubated with the reagents for 6 hours. The analyses were performed with triplicate dishes *- statistically significant *vs *MEM/BSA. **- Statistically significant *vs *TPA.

The stimulation of basal PGI_2 _production by epoxomicin and TPA-induced PGI_2 _production by epoxomicin and lactacystin as a function of dose is shown in Fig. 2. As little as 0.3 μM epoxomicin stimulates TPA-induced PGI_2 _production significantly (Fig. 2-B). It is 10 to 15 times more effective than lactacystin (compare Fig. 2-B and 2-C). Using purified bovine erythrocyte proteasomes, epoxomicin inhibits the chymotrypsin-like activity, about 4 to 5 times more effectively than does *clasto*-lactacystin β-lactone, the derivative of lactacystin [5]. They are almost equally effective on inhibiting the trypsin-like and PGPH-like activities [5]. Assuming that epoxomicin and lactacystin have equal access to the proteasome and that proteasome activity is regulating COX-2 in rat liver cells similarly to neuronal cells [12] then COX-2 may be degraded in the proteasome by cleavage after large hydrophobic residues.

The amplification of PGI_2 _production (Figs. 1 and 2) after inhibition by epoxomicin could reflect not only stabilization of COX-2 but also an intracellular increase in the concentration of the substrate i.e. the AA that is produced during hydrolysis of the membrane phopholipids by PLase activity [15]. Extracellular and/or intracellular release of AA will depend, in part, on the localization of the phospholipids in the membrane, e.g. in its inner or outer leaflet [16]. Release of AA in response to several agonists has been described [17-20].

**Figure 3:**
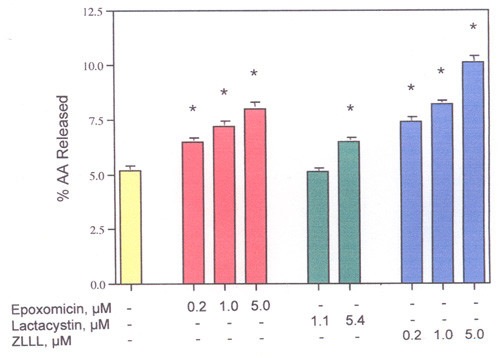
Dose-response of epoxomicin, lactacystin and ZLLL on AA release from rat liver cells. After incubation for 6 hours. The analyses were performed with triplicate dishes. *- Statistically significant *vs *MEM/BSA.

**Figure 4:**
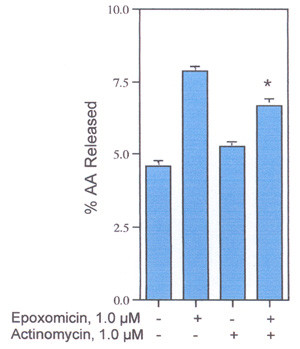
Effect of Actinomycin D on AA release from rat liver cells. Cells were preincubated with and without 1.0 μM actinomycin D for 2-h, then incubated in the presence and absence of epoxomicin and actinomycin D for another 6-h. The analyses were performed with triplicate or quadruplicate dishes. *- Statistically significant *vs *epoxomicin in the presence of actinomycin D.

**Figure 5:**
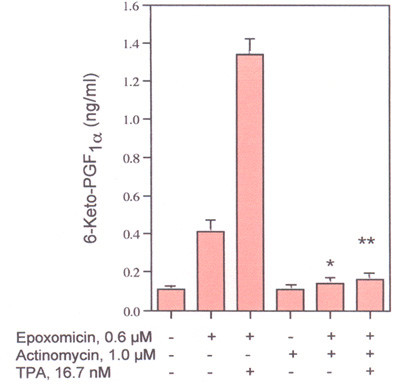
Effects of actinomycin D on TPA-induced 6-Keto-PGF_1α _production. Cells were preincubated with 1.0 μM actinomycin D for 2-h and then incubated in the presence and absence of 0.6 μM epoxomicin and/or 16.7 μM TPA for another 6-h. The analyses were performed with triplicate dishes. *- Statistically significant *vs *TPA in the absence of actinomycin D. **- Statistically significant *vs *epoxomicin plus TPA in the absence of actinomycin D.

The effect of a 2, 4 or 6-h incubation on AA release from rat liver and rat glial cells by 1.0 μM epoxomicin was determined. Only after the 6-h incubation were the differences significant statistically. Regulation of PLase activity by the proteasome pathway appears to be a relatively slow process. After a 6-h incubation, epoxomicin, lactacystin and ZLLL stimulate the release of extracellular AA from rat liver cells (Fig. 3) and AA release after TPA-induction (3.7% *vs *13.5% in the presence of 1.0 μM epoxomicin). Epoxomicin also stimulates the release of AA from rat glial, human colon carcinoma and human breast carcinoma cells (Table 1). The stimulation of AA release from the rat liver cells after incubation with epoxomicin is partially inhibited by pre-incubation of the cells for 2-h with actinomycin (Fig. 4) suggesting that a fraction of the PLase is induced. As expected, the inhibition of TPA-induced PGI_2 _production by actinomycin D is complete (Fig. 5). Thus, some mechanisms leading to maximum AA release appear to be genomic. The induced PLase activity, probably PLA_2_, could reflect expression of either a secretory or cytosolic PLA_2 _or some combination of both enzymes [21].

**Table 1:** Effect of Epoxomicin on AA Release from Rat Glial, Human Colon Carcinoma and Human Breast Carcinoma Cells.

Cell Type	% AA Release	
	MEM/BSA control	With epoxomicin
Rat glial (C-6)	9.0 ± 0.07	11.4 ± 0.28
Human Colon Carcinoma (HT-29)	7.8 ± 0.05	9.4 ± 0.33
Human Breast Carcinoma (BT-20)	12.1 ± 0.35	14.2 ± 0.31

Cells were incubated in the presence and absence of 4.5 μM epoxomicin for 6-h, 12-h or 9-h (C-6, HT-29, BT-20 respectively). Analyses were performed with quadruplicate (C-6 and HT-29) or quintuplicate (BT-20) dishes. All values are expressed as Mean ± SE (n = 4 or n = 5). All data with epoxomicin are statistically significant *vs *MEM/BSA.

The release of AA from rat liver cells, most likely resulting from PLase activation, is associated with cancer chemoprevention [14,17-19], [22-24]. In addition to its intrinsic biologic activities, AA regulates production of lipoxygenase, cytochrome P-450, and epoxygenase products as well as COX activities. Prostanoid profiles differ with cell type and individual AA metabolites have different pharmacological properties [15]. COX-2 activity, as measured by PGI_2 _production, is stimulated by proteasome inhibition (Fig. 1 and 2). Thus, some biologic effects of proteasome inhibition, e.g. stimulation of bone formation [25], may reflect the metabolism of the intracellular AA.

Inhibition of COX-2 activity is one possible mechanism that has been proposed to prevent colon cancer [26]. However, rather than inhibiting, tamoxifen and raloxifene, statins and epoxomicin stimulate COX-2 *activity *and AA release from rat liver cells [14,17-19]. As shown in Table 1, epoxomicin stimulates AA release from human colon carcinoma, breast carcinoma and rat glial cells. Tamoxifen and simvastatin also stimulate AA release from the human colon carcinoma and human breast carcinoma cells (unpublished data). These drugs have been reported to prevent cancer [27,28]. At least as measured by the COX activity of rat liver cells, tamoxifen, raloxifene, statins and proteasome inhibitors could be preventing cancer by a COX independent mechanism.

AA resulting from proteasome inhibition has many intrinsic biologic properties [reviewed in [29]]. Some of these activities may trigger PLase activity. The causal relationship of AA to cancer prevention (if any) is unclear. Production of AA by the tumor-suppressive type-II phospholipase A_2 _(PLA_2_G_2_A) may be related to the cancer prevention [22-24]. It is not surprising that control of PLase activities present an attractive area for cancer prevention studies [30].

## Methods

The rat liver (C-9 cell line) and human breast carcinoma (the BT-20 cell line) were purchased from the American Type Culture Collection (Manassas, VA, USA). The rat liver glial cells (C-6 cell line) was obtained from Dr. Elaine Lai of the Department of Biology, Brandeis University and the human colon carcinoma (the HT-29 cell line) was obtained from Dr. Basil Rigas, American Health Foundation, Valhalla, NY, USA. They were maintained in Eagle's minimum essential medium (MEM) supplemented with 10% fetal bovine serum. [^3^H] AA (91.8 Ci/mmol) was obtained from NEN Life Science Products, Inc. (Boston, MA, USA). Epoxomicin, lactacystin and ZLLL were purchased from Biomol (Plymouth Meeting, PA, USA). All other reagents were from Sigma Chemical Co. (St. Louis, MO, USA). Rat liver cells incubating with lactacystin (5.4 μM) or ZLLL (1.0 μM) for 6-h have been tested for viability by a tetrazolium-based assay and found not to be toxic [31].

Two days prior to experiments, the rat liver cells were treated with 0.25% trypsin-EDTA and, after addition of minimal essential media (MEM) containing 10% fetal calf serum, the floating cells were seeded onto 35 mm culture dishes. The plating densities varied from 0.1 to 0.5 × 10^5 ^cells/35 mm dish. The freshly seeded cultures were incubated for 24-h to allow for cell attachment. After decantation of MEM containing the fetal bovine serum, 1.0 ml fresh MEM containing 10% fetal bovine serum and [^3^H] AA (0.2 μCi/ml) were added and the cells incubated for another 24-h. The cells were washed 4 times with MEM and incubated for various periods of time with 1.0 ml of MEM containing 1.0 mg BSA/ml (MEM/BSA) and different concentrations of each compound. The culture fluids were then decanted, centrifuged at 2000 × g for 10 min, and 200 μl of the supernate counted for radioactivity. Radioactivity recovered in the washes before the incubation was compared to input radioactivity to calculate the % radioactivity incorporated into the cells [31]. For PGI_2 _production, 1.0 ml of MEM supplemented with 10% fetal bovine serum, void of [^3^H]AA, was added after the first 24-h incubation. The cells were incubated for another 24-h, washed three times with MEM, then incubated with the compounds in MEM/BSA for various periods of time. The culture fluids were decanted and analyzed for 6-keto-PGF_1α_, the stable hydrolytic product of PGI_2_, by radioimmunoassay [32].

The [^3^H] AA release is presented as a percentage of the radioactivity incorporated by the cells. Except for the time-course experiments which used duplicate dishes, three to five culture dishes were used for each experimental point. The data are expressed as mean values ± SEM. The data were evaluated statistically by the unpaired *Student's t-test*. A *P *value < 0.05 was considered significant.
